# Breast feeding practices as cultural interventions for early childhood caries in Cree communities

**DOI:** 10.1186/s12903-015-0027-5

**Published:** 2015-04-09

**Authors:** Jaime Cidro, Lynelle Zahayko, Herenia P Lawrence, Samantha Folster, Margaret McGregor, Kristen McKay

**Affiliations:** Department of Anthropology, University of Winnipeg, 515 Portage Avenue, Winnipeg, Manitoba Canada; Medical Services and Education Coordinator, Northern Health Region, 867 Thompson Drive South, Thompson, Manitoba Canada; Faculty of Dentistry, University of Toronto, 124 Edward Street, Toronto, Ontario Canada; Councillor, Norway House, Manitoba Canada; First Nations and Inuit Health, Norway House Indian Hospital, Norway House, Manitoba Canada; Family Home Visitor, Maternal Child Health, Strengthening Families Program, Norway House Cree Nation, Norway House, Manitoba Canada

**Keywords:** Oral health, Infant feeding, Aboriginal health, Early childhood caries, Breast feeding

## Abstract

**Background:**

Breastfeeding is a gift from mother to child and has a wide range of positive health, social and cultural impacts on infants. The link between bottle feeding and the prevalence of early childhood caries (ECC) is well documented. In Aboriginal communities, the higher rates of ECC are linked with low rates of breast feeding and inappropriate infant feeding of high sugar content liquids.

**Methods:**

The Baby Teeth Talk Study (BTT) is one project that is exploring the use of four interventions (motivational interviewing, anticipatory guidance, fluoride varnish and dental care to expectant mothers) for reducing the prevalence of ECC in infants within Aboriginal communities. This research explored cultural based practices through individual interviews and focus groups with older First Nations women in the community.

**Results:**

Participants in a First Nations community identified cultural based practices that have also been used to promote healthy infant feeding and good oral health. A wide range of themes related to oral health and infant feeding emerged. However, this paper focusses on three themes including: breastfeeding attitudes, social support for mothers and birthing and supporting healthy infant feeding through community programs.

**Conclusions:**

The importance of understanding cultural health traditions is essential for those working in oral public health capacities to ensure there is community acceptance of the interventions.

## Background

Breastfeeding is a gift from mother to child and considered the ideal food for infants, providing more than nutritional sustenance. The link between breastfeeding and reduced chance of early childhood caries (ECC), as well as a range of other health issues for children, is well known. Research has demonstrated that infants who are breastfed have fewer respiratory tract [1,2] and ear infections [3], diarrheal based illnesses [4], and asthma [5]. ECC are a significant concern and chronic condition amongst Aboriginal populations worldwide. The health of a child’s mouth is linked to their overall health and well-being and can have a significant impact in their day to day experiences in eating, playing, and sleeping. Infant feeding traditions such as breastfeeding in Aboriginal communities have changed in recent years as a result of larger political and social forces. There is an increasing body of literature emphasizing biomedical interventions and treatments for this condition; however little research is available on the traditional approaches of healthy infant feeding used by Aboriginal parents and caregivers. We know that the trans-generational knowledge transfer from parents and grandparents to mothers about oral health is important. Sarson and Wilson describe the role Aboriginal grandparents in teaching parents and caregivers about oral health practices such as using a facecloth to clean the gums of infants [6]. For the most part, there are significant gaps in literature around the intergenerational knowledge of childrearing practices relating to oral health for promoting healthier infant feeding and overall improved oral health in Aboriginal communities. The aim of this research is not only to contribute to already existing literature, but to highlight the importance of trans-generational knowledge transfer of oral health and infant feeding practices in Aboriginal communities. This paper focusses on three themes including breastfeeding attitudes, social support for mothers and birthing and supporting healthy infant feeding through community programs. A discussion of the literature supporting these topics is provided, highlighting the relationship of ECC in First Nations communities for infant feeding traditions and breastfeeding.

### ECC & First Nations Communities

The rates of ECC have increased dramatically. Communities, parents and governments are increasingly burdened with the social, economic and personal costs associated with treatment. ECC has a large impact on the quality of life, not only for the child, but also the family and community. In many cases, treatment requires dental surgery which means children are sedated under costly anesthetic which carries its own set of health risks. Other factors are also linked to ECC included otitis media [7] as well as poor nutrition and childhood obesity. Nutrition and obesity are also linked to co-morbidities such as Type II diabetes, which are experienced by Aboriginal adults and children at much higher rates than other Canadians [8].

ECC research in northern Manitoban First Nations communities has been underway for several years [9,10]. Research in First Nations children’s oral health within a northern context has also been underway in communities in Northwestern Ontario, who have a similar population facing similar challenges [11,12]. First Nations communities in Manitoba face some of the highest rates of ECC in Canada, with a prevalence of more than 90% in 3 and 5 year olds, with decay rates of 13.7 ± 3.2 [12]. Manitoban First Nation communities experience long waiting periods for pediatric oral surgery, which continues to impact the quality of life of children [13]. When a child resides in a remote community, the economic and social burden of flying the child and chaperones to a referral center for treatment is high. For example, the average treatment cost for ECC cases involving general anesthesia in remote communities, such as the Sioux Lookout Zone (Ontario), is estimated to be CAD $2,642.50 [11] and CAD $3,200 in Manitoban communities [13]. For these reasons, recent attention into reducing the amount of ECC in northern, Aboriginal communities has resulted in several efforts to address this issue in a way that is culturally appropriate and efficacious.

### ECC and infant feeding

The term baby bottle tooth decay is often synonymous with ECC, highlighting the degree to which dietary practices are a significant cause of the disease, specifically through the use of the bottle containing sugary liquids [11] as opposed to breastfeeding. Infant feeding is not the only factor contributing to ECC in Aboriginal communities, and a range of socio-economic factors also impact the prevalence of the disease [14]. The persistence in First Nations populations of prolonged bottle feeding as well as sugary liquids in bottles is well discussed and research on interventions shows that education-based interventions such as anticipatory guidance [15] and motivational interviewing [16] provide some reductions in the rate of ECC. Intervention-based research has also shown a decrease in the “social acceptance of prolonged bottle-feeding by tailoring the intervention to the cultural beliefs of the community so as to make people more receptive to behavioral change” [11].

It is evident the relationship between ECC and infant feeding decreases in prevalence and severity with breastfeeding up to and past twelve months [17]. We know there is a direct causal relationship between infant feeding practices and the rate of ECC in First Nations populations specifically. Part of this is due to the fact that First Nations women are less likely to practice breastfeeding [18]. Internationally, the World Health Organization (WHO), the Canadian Pediatric Society (CPS) and other groups have promoted the practice of breastfeeding, particularly in the first six months of life [19]. This is an important health priority amongst people occupying lower socioeconomic status, as breast milk is free whereas infant formula is not only expensive but requires additional resources such as sterile bottles and boiled water. In many First Nations communities’ access to clean water can be a challenge due to the lack of infrastructure and contaminated waters.

### Infant feeding traditions and breast feeding in aboriginal populations

In remote, rural communities “breast milk is the most secure and economically advantageous” way to feed infants [20] (pg. 41). Breast milk is an important aspect of food security in communities where access to market food is economically prohibitive and access to traditional country food is a challenge due to environmental contamination, decline of species and costs of procurement and availability of hunters in the family [20]. Inuit women on income assistance who would benefit most from breastfeeding due to the high costs of milk and food in northern Canada were not more likely to breastfeed because of their household food insecurity. McIsaac et al. [21] indicates there was no association found between household food security and breastfeeding in the Nunavut Inuit Child Health Survey. Decision making around breastfeeding must be considered in the larger cultural context of the loss of infant feeding traditions [21] for Indigenous women. Long standing policies and programs that have attempted to assimilate Aboriginal children such as residential schools have had negative impacts for childrearing practices. The impact of residential schools resulted in generational disconnect which inhibited the transfer of “traditional child rearing and infant feeding ways” [22] (pg. 55). Traditional childrearing practices such as infant swaddling, introduction of country foods and traditional medicines have been shown to be still in practice [23], there is no doubt that the larger social changes such as residential schools continue to impact childrearing practices. In the mid-1900s, there was a gradual move away from breastfeeding in favour of bottle feeding worldwide. This related to concerns over insufficient caloric intake for babies, the physical burden of breastfeeding for women (weight loss and fatigue) as well as the influx of women entering the workforce [24]. Related to this was the medicalization of childbirth experienced by women during the early part of the 1900s [22]. Since the 1970s, women from remote, rural communities in Canada have been required to evacuate to tertiary referral centres. This mandatory policy has shown a cascade of negative social consequences including decreased breastfeeding [25]. Other research (Veile & Kramer, [26]) conducted in Mexico focusing on Indigenous women in remote, rural settings demonstrate a connection between transitioning birthing locations and breastfeeding patterns, their findings confirm decreasing durations of breastfeeding, however intensive and prolonged breastfeeding is still the “cultural norm” and there are no taboos which prohibit breastfeeding in public.

The move towards the convenience associated with bottle feeding resulted in a decline in the practice of breastfeeding for several more years until the mid-1900s where a resurgence of breastfeeding occurred. This return to breastfeeding reflected a disillusionment towards the actual conveniences associated with bottle feeding as well as a concern for the over medicalization of feeding babies [24]. Aboriginal women and their babies however did not return to breastfeeding at the same rates as other non-Aboriginal Canadians and rates continue to remain low. In Manitoba First Nations, there has been a steady decline in breastfeeding and recent statistics indicate that from 2002/03 to 2008/2010 there was an 11.1% reduction (54% to 42.9%) in whether a child was ever breastfed at any point [27]. This is much lower than the provincial statistics which indicate that 80% of Manitoba women initiate breastfeeding, although only half are still breastfeeding after the age of six months [28]. The Chief Public Health Officer’s Report on the Health of Manitobans also notes that “breastfeeding” rates are lower in the north and in low-income areas” [28].

Martens [18], discusses the importance of knowledge and attitudes around breastfeeding amongst First Nations adolescents in Manitoba. After the delivery of the education interventions, the young First Nations female participants showed a large increase in breastfeeding beliefs and a large decrease in bottle feeding beliefs reflected in a small to medium increase in breastfeeding attitudes. This type of intervention identified the importance of adolescent breastfeeding education which would result in youth who “were more aware of the positive benefits of breastfeeding, less embarrassed about seeing women breastfeed in public, and more willing to encourage and support people in the choice to breastfeed” [18] (pg. 254). Oneha and Dodgson discuss the role of social support for Native Hawaiian mothers as being critical in not only initiating breastfeeding, but persisting. These authors [29] describe the traditional practice of breastfeeding amongst Native Hawaiians as a natural expectation, so women and people observing a woman breastfeeding was not unusual. They also describe spiritual components to breastfeeding such as rituals and prayers to improve lactation which functioned as a source of reassurance [29]. Oneha and Dodgson used a qualitative research method providing a rich description of the complexities of breastfeeding in Indigenous communities [29]. Dodgson et al., [30] describe the role of culture as being directly related to decision making around breastfeeding. They note that the decision to breastfeed as related directly to the mother’s immersion in traditional culture. If the mother is fully engaged and immersed in her traditional culture, the more likely she is to breastfeed.

### Social and cultural context of infant feeding

The decision to breastfeed is not one that always rests solely with the breastfeeding mother and in fact are influenced by the social context of the women and her family. As Hoddinott & Pill [31] describe, factors such as “smaller family size, more women working, and increasing geographical separation of families” to result in decreasing opportunities for exposure to breastfeeding” (pg 33). Other larger social changes occurring in communities such as environmental degradation and the loss of community can result in women feeling disempowered. Tait Neufeld [32] describes the impact of social and environmental disruptions associated with Manitoba First Nations communities, compounded with food insecurity results in “less perceived autonomy or self-determination as caregivers and providers” (pg 41). This is in stark contrast to the confidence and self-reliance that earlier generations of women would have experienced as resourceful providers who were held in high esteem in the family and in the community. The role of the mother and maternal leaders in the family is essential in transmitting maternal knowledge around feeding practices and larger social, economic and environmental changes have impacted this intergenerational transmission of maternal knowledge [32]. In Manitoba, the Assembly of Manitoba Chiefs administers the Maternal Child Health Strengthening Families (MCH-SF) program have a mandate which focuses on education, employment, ecological activities, culture/traditions and lifelong learning as a resource. This program has been operating in Norway House Cree Nation as one of the sites, and includes the use of local culture including traditional childrearing activities as part of their programming [33].

While research on breastfeeding and proper infant feeding describe the benefits to reduction in ECC and overall improvements to maternal and infant health, they are done so with little reference to the cultural and traditional knowledge supporting these efforts. Prevention of early childhood caries and traditional cultures in First Nations communities are considered to be separate, or culture is an “add-on”. What the literature fails to describe is how infant feeding practices, including breastfeeding are a part of the larger maternal Indigenous knowledge transmission process that can aid in promoting healthy infants including oral health.

## Methods

Research in First Nations and Aboriginal communities in generally requires the establishment and maintenance of trusting relationships [34,35]. A research relationship was already established with the Norway House Cree Nation Health Division and the investigators as a part of the BTT study. Based on the discussions of interim BTT study findings, and some of the conversations between the CRAs and research participants in the community on traditional cultural approaches, a further research partnership was created with Norway House Cree Nation Health Division. This topic required a qualitative approach and involved interviews and focus groups. Using a participatory research approach, we engaged in a partnership with the Norway House Cree Nation Health Division who assisted with the development of research questions as well as identifying research participants. A local community member was hired who was also one of the CRAs for the BTT study. During two one week intensive periods, a total of twenty interviews were held and four focus groups. The participants were primarily grandmothers and mostly great grandmothers, some of whom were former and current primary health care providers in various capacities, both in traditional health as in the biomedical field. Preliminary findings were presented in large and small group formats as well as one-on-one with stakeholders in the community. The feedback from these presentations assisted with the analysis presented in this paper. Several community members provided in depth contributions and analysis and are included as co-authors. This was an important step in ensuring that the analysis and discussion of the findings was done within the context of the community. Knowledge transfer continues to take place as we work towards developing community-based intervention programming in the various mother/child/family programs in the community.

Some principles of grounded theory [36] methodology were used in this project because this approach is particularly suitable when little is known about a topic such as is the case for Indigenous approaches for ECC prevention. Grounded theory is an approach in which the collected data is the foundation of the theory in which leads to the development of concepts. Grounded theorists start with “data and construct these data through observations, interactions and materials gathered about the topic or setting” [37] (pg. 3). Indigenous research methodologies, specifically relational accountability as described by Wilson, provided an important methodological framework for understanding relationality between the researcher, participant, community and subject, this methodology is particularly suitable given most of the authors of this project are First Nations [38]. Grounded theory and some of the Indigenous research methodologies [34,35,38] are closely aligned because they highlight the process of inter-relationality. More recent debates around the role of grounded theory in social justice research are supported [39].

Interviews and focus groups were audio recorded, transcribed and coded using NVIVO software. Beginning early in data collections using the process of coding, we sorted, separated and synthesized data at which time analytic questions were posed to the participants and community partners which allowed for fuller distillation of themes [37]. After a draft set of themes were identified the researchers returned to the community to share preliminary findings with stakeholders and research participants. Further themes were identified based with a fuller analysis with participants and community partners.

The following research questions were asked during the focus groups and interviews including: What was the process of childbirth like in Norway House in the “old days”? What were the various roles that community members and families had in supporting the arrival of new babies? How were mothers encouraged to breastfeed? Was breastfeeding considered important? How did parents deal with infant teething? What approaches were used to ease the discomfort of teething? How were the infants’ mouths cleaned? How has the process of childbirth changed in the community? How has the process of infant feeding and dealing with teething changed in the community? Why do you think tooth decay is so prevalent in young children in Norway House? Interviews were primarily conducted in English, although in some cases the participants chose to speak in Cree, in which case a translator was used. The local CRA was well known in the community as working in the area of maternal and children’s health in various programs, she was able to solicit participants through her own networks, as well as by using the local radio station. A First Nations undergraduate student from the local university also participated in gathering the data, conducting analysis and the writing of this paper.

### Ethics approval

This research was approved under the University of Winnipeg Human Ethics Review Board. In addressing the Tri-Council Policy Statement on Research Involving Aboriginal Peoples (Chapter 9), this research took a community engagement approach with the local Health Division as the authority in not only approving the research design and tools but also as collaborators and co-authors [40]. The Health Division assisted in the development of the research questions and research plan. Participants were provided with an overview of the project, how the research was intended to be used at the local level and academically, and the measures that would be taken to ensure that their interviews would be confidential (such as not using names, removal of identifying information). Participants read and signed consent forms. A translator was used when required.

## Results

My granny used to tell me…you see that glass? That’s how precious a child is. You don’t want to drop that glass to break it and you don’t want to do that to your child. This is so precious; don’t ever leave your children. Just to go out and do something else. She said make sure your children are at home. Take care of them, feed them, and put them to bed. That’s what she used to tell us.

Healthy infant feeding traditions are embedded in cultural approaches to childrearing. However, dramatic changes in infant feeding and oral health practices were identified by the research participants as being connected to larger shifts away from cultural based childrearing. The reliance on biomedical advice around infant feeding, coupled with the drastic changes in diet including the influence of processed, high sugar, high sodium food, the low prevalence of breast feeding, as well as an overall lack of education on oral health practices have shaped the “culture” of poor infant feeding and overall care for the mouths of babies in the community. These changes in infant feeding practices and oral health cannot be understood within a vacuum, and the wider social and cultural context of the community is required.

Breastfeeding and healthy teeth were seen as linked by respondents. The participants were aware that even when it was milk or formula in the bottle, improper feeding techniques and a lack of oral care resulted in tooth decay:

I see more bottle feeding than I do breastfeeding which I think should be more breast feeding. When they bottle feed, they don’t take them off until they’re 2 or 3 yrs old. It makes things worse. By the time they get off they don’t have the proper teeth, that’s when they have to go for the surgery and I see a lot of that in Norway House.

This paper focusses specifically on areas related to breast and bottle feeding practices. The primary themes identified included breastfeeding attitudes, social support for mothers and birthing, supporting healthy infant feeding through community programs, and unhealthy bottle feeding practices.

### Breastfeeding attitudes

Participants noted that breastfeeding was socially acceptable not only in the home, but openly in public during the time they were having babies. One of our oldest respondents discussed how she would openly breastfeed in public: “at the store if he was hungry I would breastfeed right there. If there was no chair I would ask for one and say “I want to breastfeed my baby”, so they would. I was not shy because everybody was breastfeeding then”.

The participants discussed breastfeeding as a natural process, which was expected and encouraged in the community. One respondent discussed the challenges of breastfeeding while working:

I breastfed all my kids and I still had a full time job. I would go home at lunch time. I would schedule everything around breastfeeding…you had to go home do it on your lunch break make sure you had, we didn’t have breast pumps back then.

Respondents noted that young women today are challenged with social attitudes in the community that do not promote breastfeeding. One respondent discussed the challenges trans-generational teaching of traditional values and childrearing to her children and grandchildren:

They said that they didn’t like the feeling of breast feeding; it hurts. They tried. It only hurts for a while. It don’t care how I explained things to her, they’ll say “mom this is 2000 it’s not those days” so if I talk to them about the old ways, it’s not the way it is now. A lot of our young people don’t want to follow the traditional way they want to go this way. Just like me being a traditional woman I go teach them, they won’t listen but if it was a white woman teaching they would listen.

Physical space in the public community to breastfeed was also identified as a barrier. There are no designated areas in the community for breastfeeding mothers. Locations such as the mall, the recreation centre, and even the clinic does not have areas designed for mothers who wish to have some private space to breastfeed. This is a particular challenge for women who feel a sense of shyness or need a private space to relax to breastfeed.

### Social support for mothers and birthing

Like many women living in remote, rural Canadian communities, the women of Norway House are not able to deliver their babies in their home community and are evacuated to Thompson or Winnipeg. Until the 1970s, women in Norway House were able to birth at the community hospital unless they were assessed as a high-risk pregnancy or delivery, at which time they were subsequently evacuated to a tertiary center for care. In recent years, the provincially funded Kinosao Sipi Midwifery Clinic has operated within the hospital and serves women during the pre and postnatal periods and arranges transportation to the tertiary center for clients.

Birthing and breastfeeding was indelibly linked for the participants. The majority of the respondents gave birth prior to the mandatory evacuation policy and was able to give birth in the community close to their spouses, children and family. Respondents discussed the role of support for new mothers from family members as being an essential aspect of healthy infant feeding and subsequently good oral health. Some women described being well supported when they were having children, and received advice on breastfeeding and other aspects of childrearing. One respondent discussed this “I think breastfeeding came from my grandmother. My grandmother always instilled in us that breast feeding was the best way”. Another woman (humorously) describes how she shares the importance of breastfeeding with young women “they (Elders) always wanted the mothers to breast feed their kids. That’s why god gave you those things (breasts)! Those are not play things! That’s how they explained it to you; for your babies not for your husbands!”

Mentorship from Elders including grandparents, aunties and mothers was important for not only practicing healthy child rearing methods, but also for social support. In the childrearing days of the participants we interviewed, women were living with extended families which enabled a supportive environment for childrearing. One participant describes this “in those days we lived with families. We didn’t have a home. We had to live with my mother in law. She did a lot of teaching on how to look after my children when they get sick. …they were my great teachers.” Another respondent discussed the role of her aunties: “That was the roles of my aunties. They taught me how to take care of my babies, how to feed them, how to not to put too much stuff so they won’t get stomach aches. They used to teach them how to give them water but while breast feeding. They told me I didn’t need to give them anything but my breast”. Another participant described this support: “There’s lack of knowledge I guess because at my age at the time I was having babies I had support from older ladies that have knowledge of breastfeeding”.

Several respondents discuss the role of their in-laws in supporting her during pregnancy and as a mother: “my father in law always used to make fire, make sure everything was ok for me. Being pregnant every year wasn’t easy. He was very supportive even though he was an alcoholic. He made sure I wouldn’t run out in things, he was always there for me”. One respondent discussed a technique of applying pressure to her breasts, which was taught by her grandmother:

My grandmother taught me how to tie myself. My grandmother would make me those things you wrap around, a belt; so you don’t get so engorged. Then you always had to learn when to drink because you don’t drink at a certain period until you’re ready. You learn all that.

Spousal support was also noted by participants as being the key to healthy infant feeding. One respondent describes this: “I had a lot of support by my husband. He was always there. When I needed to sleep he would be the one….he basically stayed home. That makes a big difference within the dad.” Another respondent discussed a similar “my husband was a family guy. He was helping me with the babies. He would hold them, and hug them. He was working outside of the home. He was gone just during the day.”

One of the respondents who lived outside of the community when she was giving birth described how her birthing experience and subsequent hospital stay outside of the community impacted her ability to breastfeed. She related her experience to the new mothers who have to give birth outside of the community who choose not to breastfeed: “because I was young, same with these young people…no one was breastfeeding. I had four other women in the ward with me at the time and none of those women were breastfeeding.” While women are in the community, they have access to “mom and baby” programs including the Canadian Prenatal Nutrition Program (CPNP) as well as the Maternal Child Health Child Health Program – Strengthening Families (MCH-SF). While women are waiting to give birth outside of the community, they do not have access to these services and programs. The critical period of time when women give birth and are able to take up breastfeeding is interrupted by having to give birth away from primary support systems by birthing in outside referral centres. For women in Norway House, their return trip to the community from a Winnipeg referral centre takes place on a nine hour bus ride or from Thompson, a three hour bus ride, which is not conducive to breastfeeding.

Participants also discussed trying to support their grandchildren who are now having children themselves and some of the generational gaps. As one respondent described “back then you were taught to listen to what the elders tell you, you did it. You didn’t question. You just did it because that was our upbringing. You don’t talk back; you do the thing you’re told to do and that’s what I did”.

Another respondent described their situation in trying to change poor infant feeding behavior:

There are a lot of grandparents that do try and help. They get offended or they don’t like to hear it. It’s not like we’re trying to get mad at them but give them at advice like that; for the kids. Today they don’t want hear this. Sometimes I’ll have my granddaughters for 3 or 4 days, then I wouldn’t give them pop or juice, they get cranky. They go home then they’re back on the cup/bottle.

Respondents shared concerns about the oral health and overall health of new babies in the community. They felt there were many factors distracting them from caring for their infants. Issues with spouses, addictions, poverty were all identified as impacting the ability of women to properly feed their infants. As one grandmother stated “they don’t make the time. There’s less of that bond and it’s easier to just give them the bottle”.

### Supporting healthy infant feeding through community programs

Healthy infant health is promoted across many programs in the community including the Social Division which provides income assistance. Aboriginal Affairs and Northern Development Canada created a policy where bottle feeding is supported by subsidizing formula for twelve months which is utilized in Norway House. As the Norway House Social Division Manager describes:

The Income Assistance Program provides extra funds to the family where a doctor/nurse prescribes the amount of milk the baby needs in a month. We provide the total number of cans and usually we provide the most financial means for our program and provide the formula by the case. For instance a can of milk costs $3.95 a can, if the doctor states the baby needs 24 cans a month, we then calculate how much a case would cost. In total that would be $118.00 extra a month for baby formula for that baby for the whole month.

This program provides for approximately 30-40 babies per year. There are several programs in the community that are mandated to provide resources around infant feeding. The CPNP and MCH-SF have high rates of participation in Norway House Cree Nation. The Kinosao Sipi Midwifery Clinic midwife has a full capacity of clients, and infant feeding practices, specifically breast feeding is promoted. Midwifery clinic staff describes the promotion of breast feeding to expectant mothers:

I don’t know how much the doctors talk about breast feeding, but I know with the midwife, she gets a lot of calls about breast feeding, even though they are not her clients and they go to the clinic (to see the doctor). She does take the calls if they’re regarding breastfeeding. We give out a lot of information on breastfeeding. We try and promote it as much as we can.

## Discussion

Several important themes arose from this project surrounding the issue of low rates of breastfeeding initiation and duration. Women’s experiences with social support included not only spousal support, but their extended family was identified as an important component to healthy childrearing and infant feeding. Participants describe community attitudes around breastfeeding, the role of social support programs and unhealthy bottle feeding practices as all contributing to children’s poor oral health.

### Breastfeeding attitudes

Attitudes towards breastfeeding have been impacted by the medicalization of birthing including the mandatory evacuation policy for remote, rural communities. While programs do exist in the community to support women who are interested in breast feeding, the larger attitudes of the community towards breastfeeding influence a women’s likelihood of making that choice to breast or bottle feed. The respondents described the trans-generational sharing of knowledge around breast feeding and childrearing practices as being a critical component to persist in breast feeding. The role of culture and knowledge is also described by Dodgson et al [30] as being a critical factor in breastfeeding uptake and duration [30].

The lack of physical places for women to feel comfortable in to breastfeed their babies were described by participants. Providing appropriate nursing rooms in public areas in the community would likely lead to an improvement in attitudes around breastfeeding as described by Martens [18]. Nursing rooms would need to also be connected to larger breastfeeding education in the community in order to be used effectively. The result may be less embarrassment about seeing women breastfeed in public and a subsequent support of women’s choice to breastfeed [18]. As more people are seen breastfeeding in public, it becomes a normalized experience for everyone. There is an important role for health care providers around education and providing a safe space. Oneha and Dodgson also describe the importance of health professionals going beyond providing useful information and employ empathy, including “acceptance, skillful reflective listening” (pg. 86) to women who are facing challenges breastfeeding [26].

### Social support for mothers and birthing

Support and mentorship is essential for women who are initiating breastfeeding, especially if it is for the first time. Adequate support also means that women will feel more encouraged to continue breastfeeding. Oneha and Dodgson describe this cultural and emotional support as being comprised of “presence, encouragement, expertise and experience” (pg. 85) from both family members as well as those in the health field [29]. This support and mentorship also provided an opportunity for women to continue breastfeeding “I learned a lot from them: the way they did it, they changed her for me; cleaned her up and they did everything for me. This helped me to continue breastfeeding”. The use of educational interventions around breastfeeding as Martens discusses would be one way for mothers to see the positive benefits of healthy infant feeding such as breastfeeding [18]. The challenges in Norway House are that women primarily birth outside of the community in tertiary centres. During the critical times post birth when they need to be supported to breastfeed, they are in many cases alone, or have few supportive people with them. Birthing away from support systems is known to result in a low uptake of breastfeeding [25].

### Supporting healthy infant feeding through community programs

While education intervention based programs may provide for some change in attitudes around breastfeeding and a movement away from bottle feeding, it is important to recognize that the emphasis on bottle feeding are a part of a larger set of changes required. As Willows and Batal (2013) state “investments in improved socio-economic and living conditions are required to improve the health of Aboriginal infants in Canada’s north” [20] (pg 47). The proliferation of bottle feeding, though seemingly economically disadvantageous for a remote community has in fact become supported through local programs and policies such as through the Social Division. This type of supplementation provides important nutrition for infants whose mothers may not have the resources to purchase infant formula and needs to be considered within the wider social context of the community. Breastfeeding promotion must also consider the health of the mother. Many clients suffer from various kinds of addiction, as the Social Division Manager describes; “in some situations, we have recognized through case assessments and the amount of (child) apprehensions that occur”. Therefore, formula is the best healthy choice for that child”. Consistent with the work of Willows and Batal (2013), it is important that any interventions that attempt to deal with infant health issues and caregivers must reflect the reality of communities [20]. Policies that are shaped by research must “help create and sustain supportive food and geographic environments essential to make it easier for Aboriginal parents and caregivers to make healthy food choices for themselves and their children” [40] (pg. 47). Local policies and programs must reflect the needs of communities directly and be culturally appropriate in order to be effective.

Healthy childrearing practices results in healthy children and can act as an intervention for the onset of childhood disease and illness. ECC is a chronic disease that is becoming increasingly prevalent in Aboriginal communities. The high rates are related to a complex set of socio-economic and cultural challenges in communities including unhealthy infant feeding and childrearing practices. Despite these challenges the use of traditional medicines and ceremony is used to support infant health [23] which continues to influence childrearing practices resulting in strong and resilient children and families.

Infant feeding traditions like breastfeeding are known to be efficacious in reducing childhood illnesses such as ECC. A number of compounding factors such as the rising price of food, limited access to country foods due to contamination, costs of procurement and diminishing number of household hunters all point to breast feeding as the optimal source of food to reduce food security for infants in northern, remote communities. In addition, the health advantages of breastfeeding include “a lower risk of acute otitis media, gastroenteritis and diarrhea, severe lower respiratory infections, asthma, sudden infant death syndrome, obesity and other childhood diseases and conditions” [41](pg. 1) and should be considered in the larger social and cultural context of each First Nations community.

## Conclusion

The issue of healthy infant feeding traditions such as breastfeeding as a cultural based intervention for ECC prevention was highlighted by First Nations grandmothers in Norway House Cree Nation. The emphasis on solely biomedical knowledge and a reluctance to use cultural knowledge and practices has meant that new mothers have less opportunity to strengthen their cultural identity around childrearing practices which would otherwise support them in making healthy choices for baby. Three themes that emerged within the area of infant feeding traditions including: breastfeeding attitudes, social support for mothers and birthing and community program support for healthy infant feeding. While this paper does not suggest harking back to the “good old days”, what we do encourage is that community health workers consider the cultural infant feeding traditions in the community as being incorporated and supported for the current generation of mothers. We also suggest that given the large amount of knowledge of the older generations in the community, that providing opportunities for younger mothers to learn not only what these traditional and cultural practices are, but how they contributed to improved infant health (including oral health) is important for making informed decisions about childrearing. Restoring cultural childrearing skills within a contemporary context, promoting traditional knowledge such as traditional medicine and teaching transcultural skills are an essential component in rebuilding and enhancing skills and pride is a mechanism for building family and community relationships as well as intergenerational support [30]. As one grandmother stated, we need to treat our children like they are precious, breakable glass, and rally around families to support the healthy development of babies.
